# Patterns of floral nectar standing crops allow plants to manipulate their pollinators

**DOI:** 10.1038/s41598-020-58102-7

**Published:** 2020-02-03

**Authors:** Graham H. Pyke, John R. M. Kalman, Dayanne M. Bordin, Lucas Blanes, Philip A. Doble

**Affiliations:** 1 Key Laboratory for Plant Diversity and Biogeography of East Asia, Kunming Institute of Botany, Chinese Academy of Sciences, CN 650201 Kunming, PR China; 2 Department of Biological Sciences, Macquarie University, Ryde, NSW 2109 Australia; 3 School of Mathematical & Physical Sciences, University of Technology Sydney, Ultimo, NSW 2007 Australia; 4 Laboratory for Applied Science and Technology in Health, Carlos Chagas Institute, Oswaldo Cruz Foundation (Fiocruz), Curitiba, Brazil

**Keywords:** Evolutionary theory, Plant evolution

## Abstract

‘Pollination syndromes’ involving floral nectar have eluded satisfactory evolutionary explanation. For example, floral nectars for vertebrate-pollinated plants average low sugar concentrations, while such animals prefer high concentrations, perplexing pollination biologists and arousing recent controversy. Such relationships should result from evolutionary games, with plants and pollinators adopting Evolutionarily Stable Strategies, and nectar manipulating rather than attracting pollinators. Plant potential to manipulate pollinators depends on relationships between neighbouring flowers within plants, for all nectar attributes, but this has not been investigated. We measured nectar volume, concentration and sugar composition for open flowers on naturally-growing *Blandfordia grandiflora* plants, presenting classic bird-pollinated plant syndrome. To evaluate potential pollinator manipulation through nectar, we analysed relationships between neighbouring flowers for nectar volume, concentration, proportion sucrose, log(fructose/glucose), and sugar weight. To evaluate potential attraction of repeat-visits to flowers or plants through nectar, we compared attributes between successive days. Nearby flowers were positively correlated for all attributes, except log(fructose/glucose) as fructose≈glucose. Most relationships between nectar attributes for flowers and plants on successive days were non-significant. Nectar-feeding pollinators should therefore decide whether to visit another flower on a plant, based on all attributes of nectar just-obtained, enabling plants to manipulate pollinators through adjusting nectar. Plants are unlikely to attract repeat pollinator-visits through nectar production. Floral nectar evolution is conceptually straightforward but empirically challenging. A mutant plant deviating from the population in attributes of nectar-production per flower would manipulate, rather than attract, nectar-feeding pollinators, altering pollen transfer, hence reproduction. However, links between floral nectar and plant fitness present empirical difficulties.

## Introduction

That floral nectars for plants pollinated by vertebrates typically have relatively low sugar concentrations, while such animals prefer high sugar concentrations, has perplexed pollination ecologists for some time^1–4^, and been the subject of recent controversy^5–7^. Nachev *et al*.^5^ argue that bats are less likely to respond to a certain absolute difference in nectar sugar concentration between available sources when average sugar concentration is high rather than low, which leads to bats selectively visiting plants with nectar of intermediate sugar concentration, and hence evolution of plants with lower nectar sugar concentration than what the bats prefer. They suggest that this is a general phenomenon explaining observed low levels of nectar sugar concentration^5^. However, Pyke and Waser^6^ point out that plants cannot be expected to evolve traits that necessarily benefit their pollinators, argue that the approach of Nachev *et al*.^5^ is unrealistic and unable to explain observed nectar sugar concentrations, and encourage an alternative approach, which involves incorporating an optimal foraging approach to understanding the behaviour of nectar-feeding pollinators into models of plant-pollinator co-evolution.

Here we expand upon this view and present a starting point and direction for pursuing this approach.

## Background

Correlations between attributes of plants and their pollinators, known as ‘pollination syndromes’, have been detected for all aspects of floral nectar (volume, concentration, composition), but these have so far eluded satisfactory evolutionary explanation^8–10^. Nectar is predominantly a solution in water of three sugars (fructose, glucose and sucrose) generally characterised based on volume, sugar concentration (% wt/vol) and sugar composition, though it may contain other chemicals, usually in very low concentrations^11,12^. Here we focus on nectar sugar concentration and sugar composition, and do not consider other possible nectar constituents, though they may also be important^13,14^.

The rates of nectar volume and energy production per flower are positively correlated with pollinator body size, leading some to suggest that this is expected simply because large pollinators require more energy than smaller pollinators^1,3,4^. However, this premise would either require that plants evolve to benefit pollinators, which is not the way evolution generally works^1,3,4^, or would require that relatively large pollinators respond more strongly to higher-than-average encountered nectar volumes than smaller pollinators, which is neither expected nor observed^1^.

It is even more difficult to explain observed negative correlations between nectar sugar concentration and body size of pollinators, separately for vertebrate and invertebrate pollinators^1–4,15,16^, especially as pollinators of all sizes prefer relatively concentrated nectar^8,17^.

Nectar sugar composition has been related to pollinator type (e.g., bats, birds, bees, flies), supposedly reflecting dietary preferences of these different kinds of pollinator^18,19^. However, observed dietary preferences exhibited by nectar-feeding animals for sucrose, glucose and fructose are often inconsistent with the prevalence of these sugars in floral nectar of the plants they pollinate^8,20–22^.

Quantitative explanations for nectar attributes are almost non-existent^1,15,23^.

Understanding such plant-pollinator relationships requires viewing them as outcomes of an evolutionary game which leads to plants and pollinators adopting an Evolutionarily Stable Strategy (ESS). Plants and animals are involved in an evolutionary game because the fitness for an individual plant or animal will generally depend on the behaviour of other individuals, both plant and animal, of the same or other species^1,3,4^. When this game reaches equilibrium, any individual deviating slightly from the population average should have lower than average fitness and hence be selected against, and this, by definition, would constitute an ESS^24^. This ESS approach, when applied just to pollinator behaviour, is identical to Optimal Foraging Theory^1,3,4,23,25^.

Floral nectar can potentially act as either ‘attractant’ or ‘manipulant’ of a plant’s pollinators^15^. If nectar affects pollinator initial visitation or revisitation frequency, independent of other cues, then it can be considered to act as an ‘attractant’ (positive or negative)^15,26^. If pollinator behaviour during or immediately following a plant visit is affected by encountered nectar, the plant can be considered to be ‘manipulating’ its pollinators^15,27^.

However, plant attraction of pollinators through nectar^9,15^ is likely a rare occurrence for the following reasons:

- floral nectar is rarely detectable by a pollinator at a distance;
- nectar concentration and composition cannot be perceived from a distance;
- it is generally difficult for a nectar-feeding animal to predict its future nectar yield, should it decide to return to its current plant at a later time;
- a pollinator would usually have difficulty to identify, remember and preferentially visit individual plants; and
- it is inefficient for it to bypass other plants to forage in this manner^15^.

For example, experiments demonstrating that pollinators can remember and preferentially return to locations offering high nectar rewards have generally had to rely on a small number of rewarded locations, extraordinary and unnaturally high nectar energy at reward locations, and distinct or extreme separation between reward and non-reward locations^28–32^. One study reports that nectar-feeding bats can remember and avoid up to 40 visited and non-replenished flowers in a square grid of 64 artificial flowers^33^, but this study fails to consider foraging strategies adopted by the bats, including directionality and distances of successive inter-flower movements, that could reduce, even minimise, flower revisitation^34–37^.

On the other hand, manipulation of pollinator behaviour through nectar is likely to be common because nectar standing crops in neighbouring flowers on the same plant should tend to be similar, through similar production and similar history of pollinator visitation^15^. Furthermore, an efficiently-foraging nectar-feeding pollinator will be influenced, whether to probe another flower on the current plant, on the basis of nectar obtained at the present flower^15^. Such manipulation of pollinator behaviour will affect subsequent pollen transfer, hence plant reproduction^15^, and consequently nectar evolution, as nectar attributes are generally heritable^38,39^.

To evaluate the potential for plants to manipulate the behaviour of their nectar-feeding pollinators through floral nectar, it is therefore necessary to determine relationships between all attributes of nectar standing crops for neighbouring flowers within plants, but this has not been done. Nectar-feeding pollinators can detect and respond to differences in volume, composition and concentration of nectar, with varying consequences in terms of energy and nutrition^8^, and so should respond to variation within and between plants with respect to all three nectar parameters^15^. Many studies have found correlations between nectar standing crop volumes for flowers from the same plant^40–45^, very few have reported similar correlations in terms of nectar sugar concentration^46^ or nectar composition^11,46,47^, and none has simultaneously considered all three attributes. The situation is the same for nectar production, except for one study in which volume, sugar concentration and sugar composition were simultaneously measured for nectar produced over a 24 h period, resulting in significant correlations between flowers from the same plant for these nectar attributes^48^. These attributes may co-vary, such that omission of one attribute or another may bias results and conclusions.

To evaluate the potential for plants to attract their nectar-feeding pollinators through floral nectar, it is similarly necessary, except in rare situations where nectar can be perceived at a distance, to determine relationships between attributes of nectar standing crops (i.e., volume, concentration, composition) for successive times when nectar-feeding visits to plants are likely and, between which, significant nectar production is likely to have occurred. Suppose, for example, that nectar production is mostly overnight, nectar-feeding tends to occur at dawn each day, and nectar standing crops on successive mornings are correlated either for individual flowers or individual plants. Then, given sufficient mental ability and priority access to particular rewarding flowers or plants, nectar-feeders might enhance their foraging success by preferentially revisiting these particular flowers or plants on successive mornings.

## Hypotheses and Aims

For any particular plant species, our major hypothesis is that there will be:

- Significant positive correlations, for all nectar attributes (i.e., nectar volume, concentration and composition), between nectar standing crops for neighbouring flowers on the same plant;
  In addition, our secondary hypothesis is that:
- Significant correlations between nectar standing crops for flowers or plants sampled at different times (e.g., same time of day, successive days) are possible, but unlikely.

This secondary hypothesis is more difficult to test than our major hypothesis, and proper testing it will require multiple studies similar to ours.

These hypotheses address the likelihoods of nectar attraction and manipulation through floral nectar, through evaluation of the opportunities for each, and we do not attempt here to consider what nectar attributes would be expected under nectar attraction vs manipulation. As we explain below, development of such further hypotheses will depend on how nectar-feeding pollinators respond to encountered nectar and this must await further investigation.

**Figure 1:**
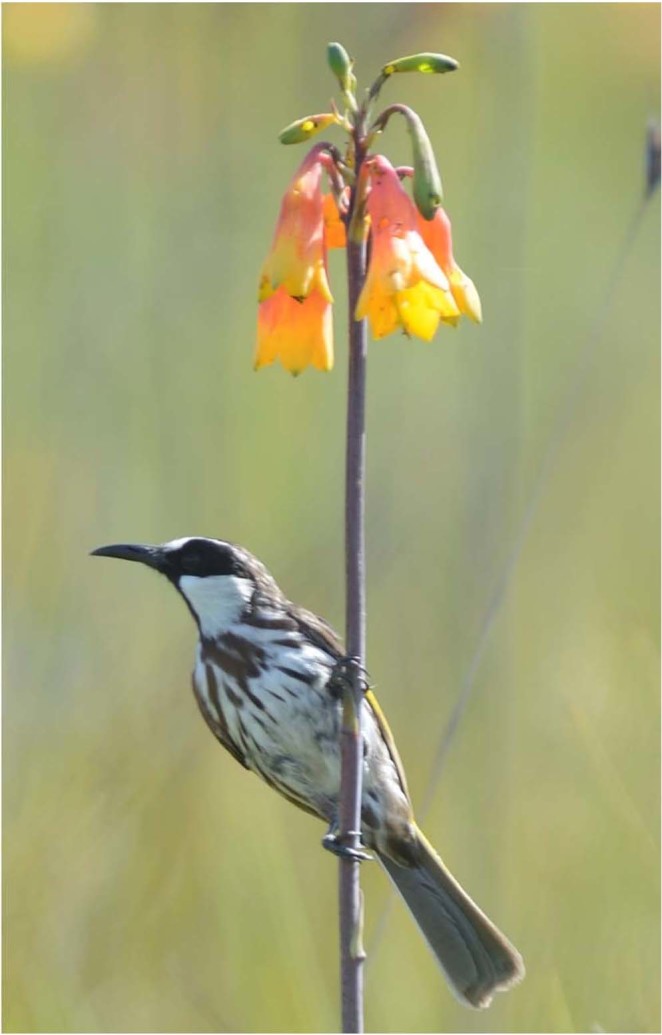
White-cheeked Honeyeater (*Phylidonyris niger*) about to begin probing flowers of Christmas Bells (*Blandfordia grandiflora*) at Kara Plantation, near Port Macquarie, N.S.W., Australia.

We evaluated the above hypotheses through sampling nectar from naturally-visited flowers on plants of *Blandfordia grandiflora*, a species that exhibits fire-stimulated flowering^49^, is visited primarily by birds, and fits the classic bird-pollinated plant syndrome, as it has relatively few large, predominantly-red flowers (Fig. 1) that produce and contain moderate volumes of relatively dilute nectar. At our study site (see below), plants of this species generally produce a single flowering stalk with about 7 total flowers on average, flowers generally have corollas that are red with yellow tips Fig. 1 ^50^, and flowers produce about 24 µl of nectar per day at an average total sugar concentration of about 17% wt/vol (unpubl.; Supplementary material, Fig. S2), all typical attributes for bird-pollinated plants^2–4^. Plants were not bagged, or otherwise covered, because we wished to sample flowers as birds would encounter them.

To consider the potential for our subject plants to manipulate their pollinators through floral nectar, and thus test our major hypothesis, we sampled nectar from all open flowers on a plant and compared nectar attributes for flowers that were vertically adjacent and hence likely to be probed successively by nectar-feeding birds. We determined nectar volume for each sample, nectar sugar concentration for samples with sufficient volume, and sugar composition for as many samples as possible (see below).

To consider the potential for birds to profitably revisit particular flowers and/or plants on the basis of nectar obtained at them, and thus test our secondary hypothesis, we compared nectar samples taken on one early morning with nectar samples from the same flowers and plants taken the following early morning. This would correspond to when the nectar-feeding birds might reasonably choose to revisit flowers or plants, as their nectar-feeding is restricted each day to a short early-morning interval, during which little nectar is produced and available nectar is depleted (unpubl.).

## Methods

Nectar was collected in an area of natural vegetation containing abundant flowering by *B. grandiflora* during November 2015, when peak flowering occurs, and during the early morning when birds were observed feeding at flowers of this species. Plants were growing on private land (Kara Plantation)  adjacent to Limeburner’s Creek National Park, about 11 km north of Port Macquarie, NSW, Australia. This property is divided into blocks measuring 150 m × 150 m and burnt on a rotational basis such that there is almost always at least one block that has been burnt within the previous 1–2 years and thus supports high densities of flowering plants of *B. grandiflora*. In this area, *B. grandiflora* flowers mostly during the period November to January. Plants were selected within one such block by walking through areas of relatively high densities of flowering plants and choosing plants as encountered that had at least two open flowers on a single flower stem, and for which no flowers (including buds) exhibited any apparent deformity. Nectar was sampled between 06:00 and 09:00 which coincided with the daily times when birds (i.e., white-cheeked honeyeater *Phylidonyris niger*) were observed visiting flowering plants and probing flowers (Fig. 1). During this period air temperatures generally ranged from about 17 to 21 °C. When the same plants were sampled on successive days, they were sampled in the same order and hence at close to the same times each day.

Nectar was sampled from each open flower on a plant, leading to estimates of nectar volume, sugar concentration and sugar composition for each flower. We sampled an average of 3.1 flowers per plant (i.e., 223 flowers across 73 plants). As flowers occur at distinct locations along a vertical stem (Fig. 1) and open successively from bottom to top, we numbered flowers successively on each plant starting with the lowermost and proceeding upwards. Birds generally approach flowers from below, probing successively higher flowers (Fig. 1). Nectar was sampled by probing each flower with a 30 µl micro-capillary tube (total length 78 mm), measuring the lengths of nectar in the tube in order to determine nectar volumes (n = 223). Nectar sugar concentration (wt g sugar/100 g nectar) was measured by placing a small amount of nectar (i.e., about 1–2 µl) in a hand-held refractometer (Bellingham & Stanley, adjusted for low volumes) and converting these measurements to wt g/100 ml using the formula Y = 0.226 + 0.937X + 0.00585X^2^ see^51^ where X is wt g sugar/100 g nectar and Y is wt g sugar/100 ml nectar, which is equivalent to wt mg sugar/100 µl nectar. Given the relatively small range in air temperatures during nectar sampling, any effect of temperature on estimated sugar concentration would be small and no more than about 0.3 wt g sugar/100 g nectar^52^. Nectar volume was sufficient for sugar concentration to be determined in 207 of the 223 cases. The remaining nectar from each flower was transferred to a small, sealed vial which was kept cold in a container with ice for about 2 hr, then moved to a freezer for 1–2 weeks, then transported on ice for about 7 hr to another freezer, where it awaited chemical analysis. Total nectar volume for each flower was determined by adding together all the capillary lengths and converting the resulting length into volume.

The concentrations of fructose (F), glucose (G) and sucrose (S) in 203 nectar samples were determined using a recently optimised method involving capillary electrophoresis and indirect UV detection. This method separated these three sugars in less than 15 minutes per sample using an electrolyte solution (i.e., 15 mM of sorbic acid, 0.5 mM CTAB and 36 mM NaOH, at pH 12). Sample preparation was made by dilution and addition of an internal standard (galactose). Calibration curves ranging between 0.1 to 4 mM were constructed to quantify all samples. Further details of this methodology will be presented elsewhere.

We characterised nectar composition for each nectar sample as the proportion by weight of sucrose (i.e., S/[F + G + S], hereafter prop S) and the logarithm of the ratio of fructose to glucose by weight (i.e., log F/G). These measures encompass all three sugars, are generally considered the most relevant ones to describe variation in nectar composition, and are identical or equivalent to those commonly adopted in other studies (see Supplementary Material).

To determine relationships between nectar attributes in adjacent flowers on the same plant, we then analysed relationships with all nectar attributes (i.e., nectar volume, sugar concentration, prop S, log F/G) for each sampled flower as independent variables, and each of these nectar variables for the next higher flower (if sampled) as dependent variable. Such assignment of independent and dependent variables is appropriate as we are interested in the potential ability of a nectar-feeding animal, that has just sampled the nectar from one flower, to estimate its likely nectar yield at its next flower on the same plant. We used a forward stepwise General Linear Model, with a threshold probability for significance of 0.015 (see Supplementary Material), and then tested for normality of residuals using the Kolmogorov-Smirnov test. We employed one-tail tests whenever the independent and dependent nectar attributes were the same (e.g., nectar volume in a flower vs nectar volume in next higher flower), since we expected and observed all such relationships to be positive. Because we used repeated data from flowers within the same plants, we included plant ID as an independent categorical variable in analyses. We carried out analyses using the software package SYSTAT^53^.

We similarly compared nectar attributes between successive sampling days for each flower and plant. In this case, the nectar per flower or per plant on one day was the independent variable and the corresponding nectar on the next day was the dependent variable. As above, this assignment of independent and dependent variables is appropriate as we are interested in the potential ability of a nectar-feeding animal, that has just sampled the nectar from a flower or plant, to estimate its likely nectar yield at the same flower or plant on the next day. However, when considering successive days for each plant, we omitted log F/G as F/G was generally close to 1 for each flower. We included plant ID as an independent categorical variable when considering successive days for individual flowers, but did not do so when considering successive days for plants.

Because birds might respond to amounts of sugar encountered, and not separately to nectar volume and sugar concentration, we also considered possible relationships between flowers and plants in terms of weight of sugar per nectar sample, which is simply the product of nectar volume and sugar concentration (wt/vol). As above, we included plant ID as an independent categorical variable where appropriate.

## Results

**Figure 2:**
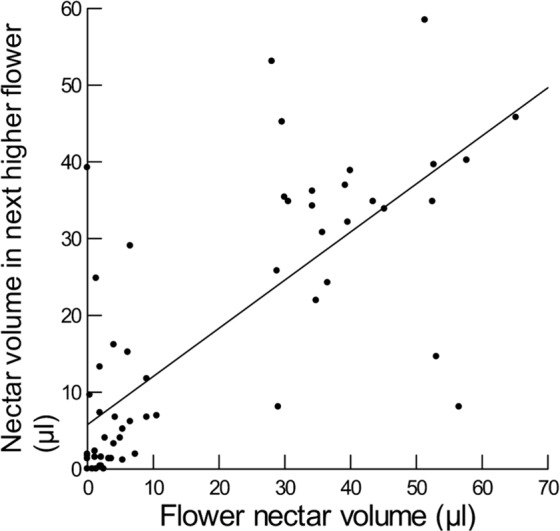
Plotted against sampled flower nectar volume (µl) is nectar volume (µl) obtained from the next higher flower on the same plant. Smooth line is fitted to these data using the General Linear Model and has equation y = 5.8 + 0.6x (R^2^ = 0.56).

**Figure 3:**
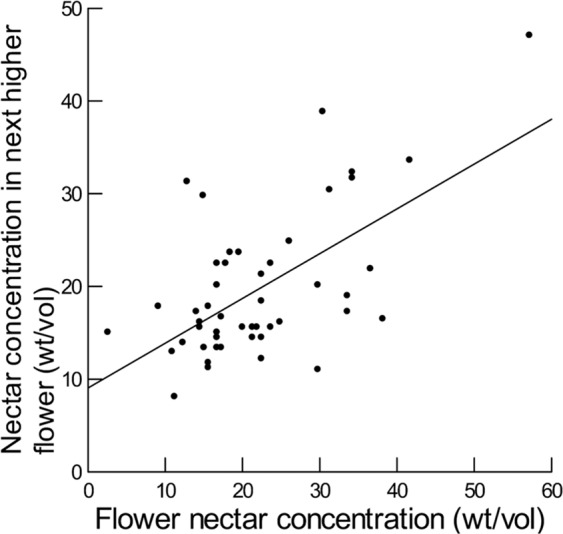
Plotted against sampled flower nectar concentration (% wt/vol) is nectar concentration (% wt/vol) obtained from the next higher flower on the same plant. Smooth line is fitted to these data using the General Linear Model and has equation y = 9.0 + 0.5x (R^2^ = 0.60).

**Figure 4:**
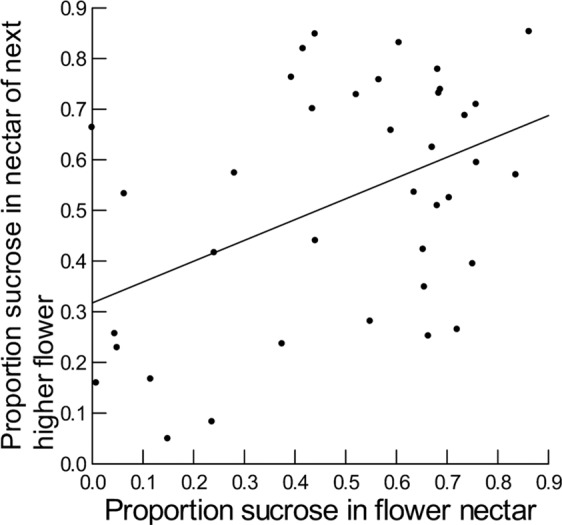
Plotted against proportion sucrose for nectar sampled from a flower is proportion sucrose obtained from the next higher flower on the same plant. Smooth line is fitted to these data using the General Linear Model and has equation y = 0.32 + 0.41x (n = 38; R^2^ = 0.20).

Nectar attributes for nearby flowers on the same plant were highly and positively correlated for nectar volume, sugar concentration, proportion sucrose, and weight of sugar, but not significantly correlated for log F/G (Supplementary Material, Table S1; Figs. 2–4). Otherwise, nectar attributes for a flower were not significantly related to any of the other attributes in the next lower flower (Supplementary Material, Table S1). In all cases, the model residuals did not depart significantly from a normal distribution (Supplementary Material, Table S1). In all cases, plant ID was not significant.

For nectar attributes for the same flowers on successive days, none of the relationships was significant (Supplementary material, Table S2). Additionally, in all cases plant ID was not significant.

For average nectar attributes across flowers for the same plants on successive days, there were significant positive relationships for average nectar sugar concentration on day 2 and both the average sugar concentration on day 1 and the average prop S on day 1, but none of the other relationships was significant (Supplementary material, Table S3).

## Discussion

We confirmed our major hypothesis that there will be significant positive correlations, for all nectar attributes (i.e., nectar volume, sugar concentration, sugar composition and sugar weight), between nectar standing crops for neighbouring flowers on the same plant.

Our study therefore reveals, for the first time, that based on nectar obtained at its present flower, a nectar-feeding pollinator could predict all nectar attributes (i.e., volume, sugar concentration, sugar composition, sugar weight) at its next flower on the same plant. In general, the next flower that it visits on its current plant will most likely be one that is relatively nearby^54–56^. In our situation, nectar-feeding birds mostly probed successively higher flowers, for which the nectar contents were highly and positively correlated in terms of volume, sugar concentration, sugar composition (i.e., prop S in our case), and sugar weight.

Furthermore, the lack of significant correlation in terms of log F/G was not surprising, given the generally very similar concentrations of F and G, and consequently low variance in the F/G ratio. In addition, this low variance in F/G would still mean that a nectar-feeding pollinator could accurately predict the F/G ratio at its next flower, and we expect that this will also be true in situations where the ratio varies more.

A nectar-feeding pollinator that has just probed a flower, especially if foraging optimally, should therefore base its decision, whether to visit another flower on the same plant or leave for a new plant, on all attributes of the nectar just obtained. Such pollinator decisions, repeated over one or more foraging bouts, will influence how much nectar is accumulated and its sugar concentration, and hence overall energy gain for the animal, as well as the composition of this nectar. These attributes of accumulated nectar, acting in combination, are likely to influence biological fitness of nectar-feeding animals and this combination would thus be the ‘currency’ by which the outcomes of alternative decision-rules are determined. A nectar-feeding pollinator, if foraging optimally, would be expected to adopt decision rules that maximise this currency, subject to constraints.

Indeed, there is ample evidence that the foraging behaviour of nectar-feeding animals while visiting natural plants is affected by nectar volume, sugar concentration and sugar composition, but little attempt to distinguish the effects of these attributes, and little or no indication regarding how they combine to influence such behaviour. For example, many studies have demonstrated that a nectar-feeding animal is more likely to probe another on a plant with increasing nectar volume obtained from the present flower^41,45,57^, but no similar study has considered nectar concentration or composition. Furthermore, many studies have also considered the effects on pollinator behaviour or plant reproduction of artificially increasing floral nectar volume with sucrose solution of a particular concentration^58–60^, but few have considered possible synergistic effects of different nectar volumes and concentrations^61^, and none has attempted to maintain constant nectar composition or varied nectar composition in any particular way. Few studies have considered effects on pollinator behaviour of nectar removal^62^. Apparently, no study has considered possible interactive effects on pollinator behaviour at natural plants of nectar volume, concentration and composition. However, some studies have considered how nectar volume, sugar concentration and sugar composition may combine to affect nectarivore feeding at artificial flowers^17,21,63^.

Regardless of how nectar-feeding pollinators respond to nectar volume, concentration or composition, or some combination of these attributes, it is clearly possible for a plant to manipulate such pollinators through adjusting its nectar attributes. Floral nectar volume will be determined by rates of nectar secretion and resorption, and removal by flower visitors. Nectar concentration will be determined by concentration of nectar as secreted and subsequent changes through resorption and evaporation. Nectar composition will be similarly determined by what is secreted and any post-secretion changes, such as hydrolysis of sucrose to form glucose and fructose. In other words, a plant has significant, though not complete, control over all attributes of its floral nectar. Because of the positive correlations between adjacent flowers for these nectar attributes, they are all likely to influence, and thus manipulate, pollinator behavior.

However, to evaluate the extent to which plants are manipulating pollinator behaviour through nectar production, further quantitative research will be necessary on how plant nectar production influences the nectar encountered by pollinators and how the pollinators respond to variation in encountered nectar. As mentioned above, the nectar encountered by pollinators should depend, at least in part, on aspects of its production. In addition, for example, experimentally modifying the nectar encountered by pollinators and observing pollinator responses should indicate how plants influence pollinator behaviour through their nectar. It should similarly be possible to determine how pollinator behaviour affects pollen transfer and hence plant fitness. Combining these processes should link variation in nectar production with variation in pollinator behaviour and hence plant fitness, thus quantifying pollinator manipulation by the plants.

Our results were also consistent with our secondary hypothesis that significant correlations between nectar standing crops for flowers or plants sampled at different times (e.g., successive days, same time of day) are possible, but unlikely. Nectar attributes for the same flowers on successive days were not significantly related. Average nectar attributes for plants sampled on successive days were significantly related only for average nectar sugar concentration across flowers on the second day, which was positively correlated with both average sugar concentration and average prop S on the first day. Other relationships for plants between successive days were not significant, including that involving sugar yield per flower, which might be the most significant variable from a pollinator’s perspective.

Consequently, as expected, the plants in our study demonstrated little potential to influence repeat visits to flowers or plants, even if this were beneficial to pollinators and they could remember individual flowers or plants. As nectar attributes for the same flowers on successive days were not significantly related, there is no apparent reason for pollinators to preferentially revisit individual flowers, even if they could. As most relationships for plants between successive days were not significant, there would be little to no benefit for pollinators to preferentially revisit particular plants.

This lack of significant positive relationships between successive days, both for individual flowers and averages across flowers within plants, suggests that levels of variability in nectar production between different days, for all attributes, generally overwhelm variation in nectar production between adjacent flowers on the same plant. Further research will be necessary to evaluate these different sources of variation.

Furthermore, a plant is unlikely to attract repeat pollinator visits through its nectar attributes, as nectar-feeding pollinators would be unlikely to benefit from such behaviour and to be capable of the necessary mental gymnastics^15^, and our results were consistent with this expectation. The nectar obtained by a pollinator during a repeat visit to a flower or plant would have predictably low volume if the interval between visits were short, or be poorly correlated in terms of nectar attributes with nectar obtained initially, if the inter-visit interval were long^15^. In our case, nectar attributes exhibited little to no significant relationships between successive early mornings, which would correspond to when the nectar-feeding birds might reasonably choose to revisit flowers or plants. In other words, a honeyeater would achieve negligible apparent benefit through preferentially revisiting particular flowers or plants based on encountered nectar. It would also be very difficult for a honeyeater to remember the identities and locations of individual plants from one day to the next, given the large numbers of plants available and utilised, their changing appearance from day to day as flowers open and age, and lack of landmarks. Similarly, preferential visitation by honeyeaters of individual flowers would surely be impossible.

We have therefore confirmed our expectation that, through nectar production, there is ample opportunity for individual plants to manipulate pollinator behaviour, no opportunity for them to attract initial pollinator visits, and scant opportunity for them to attract revisits.

Similar results seem likely for other plant species, including those with more flowers and more complicated flower arrangements, as nectar attributes are likely to be positively correlated for flowers that are close to one another e.g.,^41^ and pollinators are similarly likely to move between nearby flowers e.g.,^56^. These topics warrant further research.

However, as indicated above, we did not aim to develop and test predictions in terms of patterns of nectar distribution under pollinator manipulation and attraction scenarios. Instead, as explained below, we have established a base for understanding how nectar attributes might evolve.

It is now possible to see how attributes of floral nectar might evolve, thus pointing towards steps necessary to understand pollination syndromes involving floral nectar. For example, a mutant plant that deviated from the population average in terms of any attributes of the nectar it produces per flower would alter (through manipulation, rather than or more than attraction) the behaviour of its nectar-feeding pollinators, which would lead in turn to altered patterns of pollen transfer, and hence to altered reproduction. These would combine with changes in costs or trade-offs experienced by the plant, resulting in changes to net biological fitness. Under an ESS hypothesis, floral nectar would evolve to the point that the net biological fitness would be lower for any deviating mutant^1^.

Evolutionary explanations of plant pollination syndromes involving floral nectar will therefore hinge on understanding links between plant variation in nectar, pollinator responses during visits to plants, pollen transfer and plant reproduction, as well as on how responses to nectar by pollinators affects their biological fitness^1,15,23^.

However, developing these concepts will not be easy as there are several links between floral nectar and plant fitness, each one of which presents empirical challenges. It will be necessary, for example, to determine the abilities of nectar-feeding pollinators to detect differences across flowers in nectar volume, concentration and composition, how they respond to these differences, and consequent effects on their biological fitness^1^. A few studies have made assumptions or estimates concerning some of the links^64–66^, but only one study has attempted to consider all links^23^, and none has fully evaluated all of them^1^.

The present study does not explain the paradox of dilute floral nectar nor other pollination syndromes involving nectar, but it provides the first demonstration that nectar in nearby flowers on the same plant is correlated in terms of all nectar attributes (i.e., volume, sugar concentration and sugar composition), leading to a basis for understanding the evolution of these attributes through plant manipulation of pollinator behaviour, though requiring much further work.

### Statement of human and animal rights

This article does not contain any studies with human participants or animals performed by any of the authors.

## Supplementary information

Supplementary information
