# Supplementary material for: Patterns of floral nectar standing crops allow plants to manipulate their pollinators
Source: Sci Rep. 2020 Feb 3;10:1660. doi: 10.1038/s41598-020-58102-7 (PMC6997191; doi:10.1038/s41598-020-58102-7)
Supplement: Supplementary file 1 — Supplementary information [file 41598_2020_58102_MOESM1_ESM.docx]

Patterns of floral nectar standing crops allow plants to manipulate their pollinators

Graham H. Pyke^1,2,*^, John R.M. Kalman^3^, Dayanne M. Bordin^3^, Lucas Blanes^3^ & Philip A. Doble^3^

1. School of Life Sciences, University of Technology Sydney, Ultimo, NSW 2007, Australia
2. Department of Biological Sciences, Macquarie University, Ryde, NSW 2109, Australia
3. School of Mathematical & Physical Sciences, University of Technology Sydney, Ultimo, NSW 2007, Australia

*Corresponding author: Email: Graham.Pyke@uts.edu.au

Supplementary Material

Appendix I: Characterisation of nectar composition

Appendix II: Adjusted threshold P-value

Appendix III: References for Supporting Information

Appendix IV: Results of statistical tests

Appendix I: Characterisation of nectar composition

We characterised nectar composition for each nectar sample as the logarithm of the ratio of fructose to glucose by weight (i.e., log F/G) and the proportion by weight of sucrose (i.e., prop S or S/[F+G+S]), which are identical or equivalent, but preferable measures, compared with those commonly adopted in other studies involving nectar composition. Many studies have similarly used the proportion of sucrose or S/[F+G+S] ^1-3^. Some studies have characterised nectar on the basis of the ratio S/[F+G] ^4-6^, which is mathematically equivalent to S/(F+G+S), but it leads to a highly asymmetrical frequency distribution, making it less desirable. Many have used either the ratio F/G ^6^ or G/F ^7-9^, which are mathematically equivalent to log F/G, but less desirable as they too lead to highly asymmetrical frequency distributions. Surprisingly, however, we are not aware of any other study that has adopted log F/G (or its equivalent, log G/F) to characterise nectar composition.

Regardless, log F/G had a mean value close to zero (i.e., 0.08) and a very low s.e. (i.e., 0.04), indicating that the relative weights of F and G in nectar samples were generally very similar.

Appendix II: Adjusted threshold P-value

Because our analyses involved multiple tests we employed an adjusted threshold P-value of 0.015 for significance at each test ^10,11^. Application of the Bonferroni correction, without adjustment, to an experiment-wise P-value of 0.05 has been aptly criticised because of the consequently high expected rate of Type II errors ^12,^e.g., ^13^. So, in order to achieve a balance between Type I and Type II errors, we obtained these adjusted ‘comparison-wise’ P-values by applying a Bonferroni correction, but after first setting the overall ‘experiment-wise’ threshold P-value at 0.15 rather than the traditional 0.05 ^11^. In other words, these P-values were obtained by dividing 0.15 by the number of tests involved ^11^, but we set the maximum threshold at 0.05 as is traditionally done. We therefore did this for our ‘family’ of tests, corresponding to the relationships between neighbouring flowers within plants and flowers on successive days (10 tests), and so divided 0.15 by 10 to obtain a probability threshold for this group of tests. The same approach has been adopted previously ^14,15^.

Appendix III: References for Supporting Information

1 Rodriguez-Pena, N. *et al.* Factors affecting nectar sugar composition in chiropterophilic plants. *Revista Mexicana De Biodiversidad* **87**, 465-473, doi:10.1016/j.rmb.2016.03.002 (2016).

2 Stpiczynska, M., Nepi, M. & Zych, M. Nectaries and male-biased nectar production in protandrous flowers of a perennial umbellifer *Angelica sylvestris* L. (Apiaceae). *Plant Systematics and Evolution* **301**, 1099-1113, doi:10.1007/s00606-014-1152-3 (2015).

3 Rodriguez-Riano, T., Ortega-Olivencia, A., Lopez, J., Perez-Bote, J. L. & Navarro-Perez, M. L. Main sugar composition of floral nectar in three species groups of *Scrophularia* (Scrophulariaceae) with different principal pollinators. *Plant Biology* **16**, 1075-1086, doi:10.1111/plb.12159 (2014).

4 Hornung-Leoni, C. T., Gonzalez-Gomez, P. L. & Troncoso, A. J. Morphology, nectar characteristics and avian pollinators in five Andean *Puya* species (Bromeliaceae). *Acta Oecologica-International Journal of Ecology* **51**, 54-61, doi:10.1016/j.actao.2013.05.010 (2013).

5 de Vega, C. & Herrera, C. M. Microorganisms transported by ants induce changes in floral nectar composition of an ant-pollinated plant. *Amer. J. Bot.* **100**, 792-800, doi:10.3732/ajb.1200626 (2013).

6 Nocentini, D., Pacini, E., Guarnieri, M. & Nepi, M. Flower morphology, nectar traits and pollinators of *Cerinthe major* (Boraginaceae-Lithospermeae). *Flora* **207**, 186-196, doi:10.1016/j.flora.2012.01.004 (2012).

7 Aksic, M. F. *et al.* Influence of frost damage on the sugars and sugar alcohol composition in quince (*Cydonia oblonga* Mill.) floral nectar. *Acta Physiologiae Plantarum* **37**, doi:10.1007/s11738-014-1701-y (2015).

8 Anton, S. & Denisow, B. Nectar production and carbohydrate composition across floral sexual phases: contrasting patterns in two protandrous *Aconitum* species (Delphinieae, Ranunculaceae). *Flora* **209**, 464-470, doi:10.1016/j.flora.2014.07.001 (2014).

9 Lohaus, G. & Schwerdtfeger, M. Comparison of Sugars, Iridoid Glycosides and Amino Acids in Nectar and Phloem Sap of *Maurandya barclayana*, *Lophospermum erubescens*, and *Brassica napus*. *Plos One* **9**, doi:10.1371/journal.pone.0087689 (2014).

10 Wright, S. P. Adjusted P-values for simultaneous inference. *Biometrics* **48**, 1005–1013 (1992).

11 Chandler, C. R. Practical considerations in the use of simultaneous inference for multiple tests. *Anim. Behav.* **49**, 524-527 (1995).

12 Nakagawa, S. A farewell to Bonferroni: the problems of low statistical power and publication bias. *Behav. Ecol.* **15**, 1044–1045 (2004).

13 Perneger, T. V. What's wrong with Bonferroni adjustments. *British Medical Journal* **316**, 1236-1238 (1998).

14 Pyke, G. H., Inouye, D. W. & Thomson, J. D. Local geographic distributions of bumble bees near Crested Butte, Colorado: Competition and community structure revisited. *Environmental Entomology* **41**, 1332-1349 (2012).

15 Pyke, G. H., Thomson, J. D., Inouye, D. W. & Miller, T. J. Effects of climate change on phenologies and distributions of bumble bees and the plants they visit. *Ecosphere* **7**, 1-19, doi:10.1002/ecs2.1267 (2016).

Appendix IV: Results of statistical tests

Table S1: Relationships between flower nectar attributes (independent variable) and attributes in next higher flower (dependent variable), as determined by forward stepwise GLM. Coefficients are presented where relationships were significant, along with s.e. t-statistics and associated probabilities are also presented. Relationships were significant if P<0.015, which are indicated with *. Also presented are tests for departure from normality for model residuals using Kolmogorov-Smirnov test.

| Dependent variable  (Attribute for next higher flower) | N | Independent variable (Flower attribute)  Coefficient (if significant) ± s.e.  [t-statistic, P] | Independent variables (Other flower attributes)  [P’s] | Test for departure of residuals from normality  [P] |
| --- | --- | --- | --- | --- |
| Volume (µl) | 57 | Volume (µl)  0.63 ± 0.08  [8.39, <0.001^*^] | >0.08 | 0.07 |
| Conc (% wt/vol) | 48 | Conc (% wt/vol)  0.48 ± 0.10  [5.13, <0.001^*^] | >0.5 | 0.73 |
| Prop S | 38 | Prop S  0.41 ± 0.14  [3.03, 0.003^*^] | >0.3 | 0.80 |
| log (F/G) | 38 | log (F/G)  [1.10, 0.15] | >0.1 | 0.02 |
| Wgt sugar (mg) | 57 | Wgt sugar (mg)  0.005 ± 0.001  [7.39, <0.001^*^] |  |  |

Table S2: Relationships between nectar attributes for each flower on day 1 and attributes for the same flowers on day 2. Relationships were significant if P<0.015, which are indicated with *.

| Dependent variable  (Flower nectar attribute for next day) | N | Independent variable (Flower nectar attribute on initial day)  Coefficient (if significant) ± s.e.  [t-statistic, P] | Independent variables (Other flower nectar attributes on initial day)  P’s |
| --- | --- | --- | --- |
| Volume (µl) | 57 | Volume (µl)  [0.33, 0.38] | >0.1 |
| Conc (%wt/vol) | 51 | Conc (% wt/vol)  [1.00, 0.16] | >0.02 |
| Prop S | 49 | Prop S  0.43 ± 0.18  [2.42, 0.01^*^] | >0.1 |
| log (F/G) | 49 | log (F/G)  [0.51, 0.31] | >0.3 |
| Wgt sugar (mg) | 57 | Wgt sugar (mg)  [0.22, 0.42] |  |

Table S3: Relationships between average nectar attributes per flower for each plant on day 1 and averages per flower for the same plants on day 2. Coefficients are presented where relationships were significant (i.e., P<0.015, indicated with *).

| Dependent variable  (Plant average nectar attribute for next day) | N | Independent variable (Plant average nectar attribute on initial day)  Coefficient (if significant) ± s.e. [t-statistic, P] | Independent variables (Other average plant nectar attributes on initial day)  P’s |
| --- | --- | --- | --- |
| Avg Volume (µl) |  | Avg Volume (µl)  [0.30, 0.38] | All others  P’s>0.2 |
| Conc (%wt/vol) | 29 | Avg Conc (% wt/vol)  0.40 ± 0.13 [3.01, 0.003^*^] | Avg Prop S  29.6 ± 9.9 [2.99, 0.006^*^]  Avg Volume  P=0.5 |
| Prop S | 29 | Prop S  [2.03, 0.026] | All others  P>0.05 |
| Weight sugar |  | Weight sugar  [0.08, 0.47] |  |
